# COVID-19 and parents of children with epilepsy: Experiences and positive changes

**DOI:** 10.3389/fpubh.2023.1079518

**Published:** 2023-02-09

**Authors:** Flora Koliouli, Marianna Andrianakou

**Affiliations:** ^1^Psychology Department, National and Kapodistrian University of Athens, Athens, Greece; ^2^School of Early Childhood Education, Aristotle University of Thessaloniki, Thessaloniki, Greece

**Keywords:** parents, children with epilepsy, COVID-19, lived experiences, qualitative research

## Abstract

**Introduction:**

The aim of this study is to explore the ways that parents with children or adolescents with epilepsy (CAWE) experienced the restrictive measures, as well as the stressors and challenges that they had to face.

**Methods:**

We employed an experiential approach and fifteen Greek-speaking parents answered to an in-depth semi-structured interview, during the second lockdown period. Data were analyzed through the Thematic Analysis (TA).

**Results:**

The emerging themes were the challenges encountered in terms of medical monitoring, the “stay-home” impact on their everyday lives as a family, their psycho-emotional responses. More specifically, parents identified the irregular doctor appointments and their struggle to access the hospital services as the most important challenges. Moreover, parents reported that the “stay-home” impact has disrupted their children's daily routines among others. Finally, parents highlighted their emotional strain and worries experienced during the lockdown along with the positive changes that occurred.

## 1. Introduction

Recent studies examined the consequences of the COVID-19^1^ pandemic and the lifestyle modifications imposed by it had on general population (1, 2) and on patients with chronic illness and their families. Given the re-organization of healthcare services due to the pandemic, patients with chronic illness found it difficult to keeping up with regular appointments with their treating doctor (3). More specifically, due to the safety measures as well as the fear of contracting the virus, routine appointments were postponed or significantly delayed (4). Moreover, the changes in healthcare availability have resulted in the postponement of non-urgent surgical operations (3). An additional challenge concerns the disruption of the healthcare units' function, caused by staff reduction due to COVID-19 infection and the subsequent lockdown (4).

In the context of patients with epilepsy, going to the Emergency Room of the hospital can expose patients to the virus (5, 6), and thus is not recommended by the Epilepsy Foundation (3). The reluctance of persons with epilepsy (PWE) to visit the hospital due to the fear of infection might lead them to underestimating their symptoms and, as a result, put themselves at risk (4).

As illustrated above, chronic illness care is negatively impacted during the pandemic. A solution to this might be lying in the use of telemedicine as a valid alternative to conventional medicine which has been largely introduced during the pandemic. Regular appointments can take place through videocalls, and patient-doctor communication can be maintained through text messages (7). As far as patient satisfaction is concerned, according to an Argentinian study where parents of children on a Ketogenic Diet were consulting their doctor through the WhatsApp application, the vast majority were satisfied and would recommend the use of telemedicine (6). In the same line, the study of Reilly et al. indicates that young people with epilepsy (12–25 years old) and their caregivers were largely satisfied by the telehealth system. In addition, with the use of telehealth prescriptions for medication can be given electronically without requiring an in-person visit (3, 8).

Nevertheless, parents with children with disabilities, who took part in Ugandan research, they narrated that they couldn't keep up with their child's doctor appointments because of the lockdown (9). Parents with CAWE reported the obstacles they encountered during the lockdown such as their inability to reach for a neurologist during telemedicine resources, living in homes without a terrace or yard, financial issues, loss of regular stimulation and physical therapies, cancelation of essential medical appointments and difficulties finding their antiseizure medication at a pharmacy (10, 11).

As far as the psycho-emotional impact of the pandemic is concerned, it is suggested that the additional challenges that people with chronic illnesses face might exacerbate any preexisting psychological difficulties (7). More specifically, the results of a Saudi Arabian study illustrate that 60% of PWE who took part in the research, mentioned elevated stress levels after the start of the pandemic, while 30% also noticed an increase in seizure frequency. Conversely, the lifestyle modification that lockdown imposed, resulted in better treatment compliance and regulation sleep schedule, leading to better seizure control (4). Thus, it appears that seizure frequency was sufficiently managed by PWE and seizures were decreased during the COVID-19 lockdowns (4, 12). However, possible deterioration of the mental health of PWE and their families, especially as far as anxiety and depression are concerned, was also highlighted by French et al. During lockdowns, children were found to be suffering from anxiety, depression, irritability and inattention and a substantial fear of COVI-19. Children with pre-existing behavioral problems like autism and attention deficit hyperactivity disorder have a high probability of worsening of their behavioral symptoms (13). Caregivers have developed anxiety and depression, respectively, while being in isolation with children (13). Furthermore, parents of children with disabilities mentioned that they experienced stress due to the financial consequences of the pandemic (9). In the same study, some parents expressed their fear of contracting the virus. Moreover, caregivers of hospitalized children exhibited a higher prevalence of worrying, nervousness, and fear of infection compared with studies conducted before the lockdown policies (14).

Although existing literature has examined the impact of the COVID-19 pandemic on people with chronic illness and their caregivers, there is no sufficient information concerning the challenges that the parents of CAWE faced during this time. Furthermore, in Greece, severe restrictive measures were imposed from November 2021 and included, among others, the suspension of work, schools, universities and traveling, as well as a general lockdown. More specifically, remote work was established when applicable, and online education was performed for students of all grades. Given the number of students, online classes were distributed in different time during the day: for middle and high school students lasted from 09:00 to 14:00 whereas for elementary pupils, classes were performed in the afternoon (e.g., 13:30–17:00). The health system did not remain unaffected, as many hospitals turned into COVID-19 units while others were short on staff. Thus, there has been a long waiting list for medical appointments and scheduled surgeries.

###  Study design

In our study, we opted for a qualitative, experiential approach in order to explore how parents of CAWE experienced the pandemic, as well as the stressors and challenges that they had to face. Current empirical studies have explored parents with CAWE lived experiences (15–17), however the restrictive conditions that parents with CAWE had faced are not yet investigated. Caregivers of CAWE face particular challenges, thus an experiential approach investigating the lived experience (18, 19) can offer us a deeper understanding on the subject. In this article, our research question is formulated as following: “How do parents with CAWE experienced the COVID-19 restrictive measures?”

## 2. Methods

### 2.1. Sample characteristics

Participants were chosen through a convenience sample obtained by posting on social media. Participation criteria included whether individuals (a) spoke Greek, (b) were parents of CAWE, and (c) could access online social groups. The sample consisted of fifteen Greek-speaking participants: 14 mothers and one father who had a child or adolescent with onset epilepsy. Parents whose CAWE presented mild or severe epileptic seizures were also included. Their average age was *M* = 45 years old, the youngest participant being 38 and the oldest 53 years old. Their children's age was between two and 18 years old, with a mean age of 11 years. An average of 5 years had passed since their child was diagnosed with epilepsy (the shortest period was 1 year and the longest 10 years) (see Table 1).

**Table 1:** Sociodemographic data.

**Participant**	**Age**	**Child's age**	**Diagnosis**	**Family status**	**Child's sex**
Nefeli	38	2 years 6 months	West syndrome^1^	Married	Female
Melina	53	14 years	Non-specified	Widow	Female
Stavroula	51	18 years	Non-specified	Married	Male
Olga	45	17 years	Non-specified	Married	Male
Lydia	46	11 years 6 months	Febrile seizures plus^2^	Married	Male
Panagiota	36	2 years	Non-specified metabolic syndrome	Married	Male
Magda	52	18 years	Non-specified	Married	Male
Natalia	45	9 years	Panayiotopoulos Syndrome^3^	Married	Female
Alexandra	42	9 years	Drug-resistant epilepsy	Married	Male
Argyro	42	7 years 6 months	Atypical benign focal epilepsy of childhood	Married	Male
Marietta	41	7 years 6 months	Drug-resistant epilepsy and status epilepticus.	Married	Female
Konstantina	49	15 years	Panayiotopoulos syndrome	Divorced	Male
Eirini	44	15 years 6 months	Non-specified	Married	Female
Sophia	41	3 years	Edward's syndrome	Married	Female
Dimitris	42	7 years and 6 months	Atypical benign focal epilepsy of childhood	Married	Male

All names are fictional, and we have pseudonomized all personal data to safeguard participants' anonymity.

^1^**West Syndrome**, is a severe infantile epilepsy syndrome with a characteristic age of onset (2-14 months), pattern of seizures and electroencephalogram (EEG). There is high morbidity (intellectual impairment, ongoing epilepsy, etc.) associated with infantile spasms. www.sheffieldschildren.nhs.org.

^2^**Febrile seizures** or febrile seizures plus (FS+) are the most common seizure type in GEFS+. FS+ diagnosis means that the person has seizures (both febrile and not febrile) beyond age 6 years, and although seizures typically stop by adolescence, they could rarely continue into adulthood. https://www.epilepsy.com/learn/types-epilepsy-syndromes/genetic-epilepsy-febrile-seizures-plus#:~:text=Febrile%20seizures%20or%20febrile%20seizures%20plus%20(FS%2B)%20are%20the%20most,could%20rarely%20continue%20into%20adulthood.

^3^**Panayiotopoulos syndrome** (PS) starts in early childhood, usually between the ages of 3-6 years, but children from 1-13 years have been described. Both boys and girls can develop PS. It occurs in ~3 out of 50 (6%) children between the ages of 1-15 who have epilepsy. Children with PS mostly have normal physical and cognitive development. https://www.epilepsy.com/learn/types-epilepsy-syndromes/panayiotopoulos-syndrome.

### 2.2. Data collection

Semi-structured interviews occurred from February 1st, 2021, until March 8th, 2021. This period coincided with the second nationwide lockdown in Greece during which the worse rates in COVID-19 infections have been reported. During this period, the entire Greek population was requested to follow government orders and to limit non-essential movement to prevent further spread of the coronavirus disease. In some parts of Greece, there was a night curfew imposed from 18:00 p.m. to 8:00 a.m. Consequently, schools were closed, and online education was carried out. Interviews were conducted through video calls using Skype or Zoom by the same investigator (psychologist) who gathered all the data. We had not imposed a time limit and their average duration was 40 min, from 15 to 65 min.

The same interview protocol of open-ended questions was used. As this article is a part of a larger study exploring the lived experiences of parents with CAWE (16), we will present data relevant to the COVID-19 experience. A series of questions were asked to participants including their experiences (*Could you please share with us your experiences of the ongoing pandemic? Could you please describe in which ways the current pandemic has changed your life as a parent*? *Could you tell us more about the challenges you encountered during this period?*). The wording of the interviews was translated in English by the first author after the analysis and then corroborated with the second author. The act of translation needs to be identified as the researcher/ translator is inextricably bound to their socio-cultural positioning (20).

### 2.3. Data analysis

The Thematic Analysis (18) was employed for the interview analysis. According to TA, the identified themes are strongly connected to the data themselves. A thematic content analysis (interview per interview) was conducted to explore the emerging themes in the discourse of each parent on their personal experience. Initially, both authors read and re-read the interviews to be able to familiarize themselves with the data. During the following stage of the analysis, both researchers generated descriptive codes based on extracts of the data that were of interest for the study and named the generated codes highlighting the text associated with them. For instance, initial themes were developed according to participants' narratives; some of them evoked the practical issues addressing more descriptive aspects of current pandemic and others related to more psychoemotional experiences ex. “We haven't had a check-up in like a year or so, which is stressful. It's hard not getting a clear view of the epilepsy's evolution”. Then, themes were integrated across transcripts to identify shared themes that captured the participants' experiences. More specifically, according to the number of participants who addressed the same categories, some were merged with others and some themes were reorganized as they addressed the same issues. For example, initial code about “impact of restrictions” which addressed the lack of socialization and “the stay-home policy” that described the online education were then merged in one theme distinguished in three subthemes. In the fourth phase, both authors reviewed the generated themes and subthemes in relation to the entire coded data and the research questions. So, we identified areas of similarity or even overlap between codes (e.g., codes relating to the encountered challenges), and then grouped them together by subtheme and then theme. However, the theme “positive view of the situation” emerged from data themselves as there was not a question addressing this aspect. Then, we attributed names and definitions for the themes and subthemes and examined each theme's relation to other themes or subthemes. Throughout the analysis process, there was debriefing between the two researchers.

## 3. Results

Participants narrated in which ways the pandemic and the restrictive measures affected both their epilepsy management and their emotions. Participants mentioned some challenges they encountered such as medical monitoring of their child and staying at home in the context of the lockdown. In terms of psycho-emotional reactions about the virus, it seems that the participants, except for one mother, did not fear the possibility of their child contracting the virus (cf. Table 2).

**Table 2:** Emerged themes and subthemes.

**Theme**	**Subtheme**	**Indicative quotations**
Medical monitoring challenges encountered	Irregular follow-ups	“*So, about the follow-ups… although A. has had some doctor appointments from time to time to see how he is doing and to talk to the doctor, we have skipped two, because the doctor himself when we told him that A. is fine… and he hasn't shown any problem, she told us to wait*. “(Dimitris)”
	Reduced access to hospitals	“*But there is a practical issue with children in general. When they have a fever, in order to take them to the doctor, you must have ruled out that they have contracted corona. Appointments, distances, an escort, hospitals do not accept regular cases. All this makes it difficult for us*.” (Panagiota)
The stay-home policy impact	The struggle of online education	“*I am troubled and worried by the fact that he misses out on many things from school, and in the social part of course, but mainly in the learning part. At school, he has parallel support, so he has a person next to him to help him, while now at home I can't help him […]. It's definitely set him back and that's a big stress for me because he's already back*.” (Alexandra)
	Ruptures in the child's daily schedule	“*And from a logistic point of view, when children are at home and they don't go to school, that is… Well, now that we are doing the ketogenic diet, our child being at home all the time does not help, his mind is always on eating, while at school you put everything in the taper ware and off he goes and that's it. He has things to do at school*.” (Alexandra)
	Lack of socialization	“*It has affected both us and our child because he would go for a walk, we would all go somewhere together [with friends and family]*.” (Magda)
Emotional responses: strain and worry	Strain	”*But I have to tell you that as time goes by, I have to tell you that this thing has started to strain me too much because I think that we didn't have enough of everything else, we have this too. It bothers me beyond belief*.” (Nefeli)
	Stress and worry	“*I feel like kids are missing things and we're locking them in and creating a lot more problems for them than they already have*.” (Alexandra)
Positive view of the situation	Rapid medical prescription for their child's condition	“*From a medical point of view, we were relieved by all the electronic media that suddenly came into our lives during the first lockdown. And with the immaterial prescription and the fact that we can now make an electronic request for some allowances or submit the receipts from the speech therapists. Because we did all this before in person. We had to go and wait in line etc. Now it's all done electronically so in that sense it's better*.” (Dimitris)
	Perceived control over child's schedule routines	“*Just the same now because he's inside he doesn't take any more steps outside, it's probably safer for us now*.” (Melina)

### 3.1. Medical monitoring challenges

The restrictive measures have contributed to the irregularity of medical monitoring. Several participants indicated that during the pandemic period they were struggling to have their child's regular follow-ups and that when they finally could go, the conditions inside the hospital are challenging.

#### 3.1.1. Irregular follow-ups

Five participants stated that the child's visits to the doctor or hospital have decreased since the pandemic began whereas others have not related this issue.

“*So, about the follow-ups… although A. has had some doctor appointments from time to time to see how he is doing and to talk to the doctor, we have skipped two, because the doctor himself when we told him that A. is fine… and he hasn't shown any problem, she told us to wait*.” (Dimitris)

“*We haven't had a checkup for like a year now, which is an additional stress. It's one thing to go every 6 months and be told that everything is fine—of course we've had two teleconferences with our doctor where we sent her the tests—but it's a difficulty that we don't have a clear picture of the progression of the disease. This is hard*.” (Lydia)

#### 3.1.2. Reduced access to hospitals

Another issue that emerged from the narratives of some participants concerned the conditions prevailing in hospitals due to the measures to deal with the pandemic. More specifically, the implementation of measures such as the mandatory COVID-test, visiting only by appointment and being accompanied by only one guardian, despite their undeniable usefulness in safeguarding public health, seem to have made the conditions of access to the hospital more difficult.

“*But there is a practical issue with children in general. When they have a fever, in order to take them to the doctor, you must have ruled out that they have contracted corona. Appointments, distances, an escort, hospitals do not accept regular cases. All this makes it difficult for us*.” (Panagiota)

### 3.2. The “stay at home” policy impact

It is worth mentioning that two participants claimed that their lives did not entirely change since they stayed at home due to the pandemic, as the lockdown situation was already familiar to them. More specifically, they indicated that their everyday lives were already constrained due to the epilepsy seizures. As Sophia typically stated:

“*Personally, it hasn't affected me at all, and it hasn't changed anything in my life apart from wearing a mask to go to the supermarket. It has not affected me in anything else… Nothing has changed in my life. To me it's the same as it was before*.” (Sophia)

However, eight participants related to the challenges encountered due to this policy. These are online education, the changes in the child's daily schedule as well as the inability to socialize with other people and carry out social activities in general.

Regarding the online education, interrupting school and online lessons were highlighted as an aggravating factor of staying home for two participants, as their children's educational needs were not met:

“*I am troubled and worried by the fact that he misses out on many things from school, and in the social part of course, but mainly in the learning part. At school, he has parallel support, so he has a person next to him to help him, while now at home I can't help him […]. It's definitely set him back and that's a big stress for me because he's already back*.” (Alexandra)

Consequently, for some participants, the lockdown has brought changes in the child's schedule, as their daily lives were disrupted.

“*During the previous lockdown there were ups and downs. A week after the schools closed, he had a terrible deterioration. […] It had affected us all very much. When I heard about another lockdown again in the fall, I was afraid that we might have another one. But now the treatments he is doing are continuing, while they had stopped in the spring. […]. In other words, because he largely maintained his everyday routine, we didn't have the deterioration we had in the spring*.” (Argyro)

“*And from a logistic point of view, when children are at home and they don't go to school, that is… Well, now that we are doing the ketogenic diet, our child being at home all the time does not help, his mind is always on eating, while at school you put everything in the taper ware and off he goes and that's it. He has things to do at school*.” (Alexandra)

“H. *should exercise. Exercising is essential for stress management, for all of us that is, but also for H., one more reason why he needs to defuse his tension. And basketball courses have stopped since the beginning of the pandemic. I have hired a personal trainer so he can work out. I would prefer a team “thing” but since it's not possible… We do with what we have…*!” (Konstantina)

Furthermore, several participants reported that they have been burdened emotionally as they are unable to go out and socialize.

“*It has affected both us and our child because he would go for a walk, we would all go somewhere together [with friends and family]*.” (Magda)

“*It's the impact of the lockdown that… well, I used to do a lot of things, going out, socializing*” (Alexandra)

“I *feel like angry; I want to go out, go for a trip, have some holidays, meet with our loved ones, this would actually help us, my family and myself* ” (Marietta)

“*He cannot meet his friends because kids are not supposed to go out… So, they stay home and play on their computers, which I think that further influenced my son's seizures*” (Konstantina)

### 3.3. Emotional responses: Strain and worries

Several participants (*N* = 6) have been affected on an emotional level by the pandemic and the restrictive measures. Participants expressed their stress about the pandemic and their constant worries that their children are missing things in their lives due to the lockdowns.

Four participants reported that the lockdown has induced additional stress.

“*But I have to tell you that as time goes by, I have to tell you that this thing has started to strain me too much because I think that we didn't have enough of everything else, we have this too. It bothers me beyond belief* .” (Nefeli)

“*I often feel trapped*.” (Magda)

For Magda, the feeling of being “trapped” related that she has always been very active and not being able to follow her everyday routine with her family has a considerable impact on her emotional state. To manage her stress, she “*enrolled in a psychology program for everyone to pass the time. Fortunately, we live in a house*,^2^ *and I do a lot of gardening”* (Magda).

Two participants expressed their concern they have because they cannot provide their children with what they would like.

“*I feel like kids are missing things and we're locking them in and creating a lot more problems for them than they already have*.” (Alexandra)

“*It really gets me because I know that she could be more stimulated. We could go out for walks, go to the beach, go for swinging, and meet other little fellows and interact with them. I can't offer her all these…*” (Nefeli)

However, participants did not express additional worries about their children contracting the virus

“*We are not worried. For instance, earlier today, my cousin told me that X should also be careful if he has this issue. No. I refuse to see it that way. X should be careful because we have his grandparents around us, and because in any case I wouldn't want him to be the one spreading the virus somewhere else*.” (Stavroula)

“*The pandemic does not scare me, this virus for instance. It's the everyday life that has dramatically changed and that worries me*.” (Panagiota)

“*I am not afraid of contracting the virus. We are not in high-risk groups, so I am not worried by that*.” (Alexandra)

### 3.4. Positive view of the situation

Some participants, however, have identified some positive changes that have encountered since the pandemic outbreak such as having facilitated the bureaucratic system as far as the medical prescriptions are concerned.

One participant mentioned the convenience of being able to complete the necessary paperwork electronically, which was offered to reinforce social distancing.

“*From a medical point of view, we were relieved by all the electronic media that suddenly came into our lives during the first lockdown. And with the immaterial prescription and the fact that we can now make an electronic request for some allowances or submit the receipts from the speech therapists. Because we did all this before in person. We had to go and wait in line etc. Now it's all done electronically so in that sense it's better*.” (Dimitris)

Two participants reported that another positive element was that their child is less likely to go out alone and therefore they are not as worried about having a seizure somewhere outside the home.

“*Just the same now because he's inside he doesn't take any more steps outside, it's probably safer for us now*.” (Melina)

## 4. Discussion

The aim of this study was to explore the ways that parents with CAWE experienced the restrictive measures, as well as the stressors and challenges that they had to face. Main results highlighted the challenges that parents encountered in terms of medical monitoring, the “stay-home” policy impact on their everyday lives as it has disrupted their children's daily routines among others, and their emotional strain and worries experienced during the lockdown.

In our study, parents indicated the challenges in terms of medical monitoring, which has been reduced, the irregular doctor appointments and follow-ups along with their hesitation to access the hospital facilities. Our results are in line with those of Davico et al. (21) according to which, in Italy there has been a 38% decrease of emergency departments for seizure related reasons. Moreover, in recent literature, the outpatient visits for new patients were postponed, and follow-up visits mostly managed by telehealth (4). Indeed, it is suggested that telemedicine is feasible to conduct with children with epilepsy (6), and young people and their caregivers appear to be satisfied by telehealth (8). However, in our sample, telehealth has not been proposed, leaving families unattended for a long period of time. This finding is consistent with those of Brambilla and coll, according to which, families with CAWE would have appreciated greater use of remote consultations with their medical teams because half of clinic appointments were postponed (22).

Parents reported that their lives have been largely disrupted by the restrictive measures. The “stay-home” policy had a large impact on their child's daily routine: online education and inability to leave the house premises have put an additional strain on these children and their families. According to our sample, parents confronted challenges to adhere to their child's ketogenic diet because their children could not follow their regular routines. Our findings are contradicting those of Ferraris et al. as in their study, participants complied to the dietary regimen (23). However, their sample consisted of adults whereas, in our research, compliance to ketogenic diet concerned underaged children who might still struggle to comply.

As far as school activities were concerned, participants pointed out the struggle that online education may induce for their children such as difficulties in following the school program and disrupting their everyday routines. These findings are line with current literature, according to which 39% of patients presented difficulties in following provided school program, manifesting attention, and homework organization problems (30%), thus requiring an increased parents' assistance. Patients showing major difficulties related to homework organization and adherence to provided school program, as predictable, belonged to the group with neurocognitive comorbidity. This reflects the difficulty of coping with new learning setup, without usual supports, especially for patients for whom school functioning represents a critical issue independently from COVID-19 conditions (12, 24).

Another impact of the stay-home policy has been the lack of socializing. Although existing literature points out the impact on the social lives of families with CAWE, reduced socializing and alterations in their social schedules, it also noted their need of social support as crucial (25, 26). In our study, families with CAWE expressed the impact of not leaving the home premises and socialize with others. They have also noted that their children have been socializing online which they feared that it might aggravate their seizures' status. It seems that the restrictive measures due to the COVID-19 pandemic have triggered our participants' need to reestablish social connections and regular activities such as traveling and meeting up with friends and peers.

Surprisingly, parents in our sample were less worried about their children contracting the virus, controverting existing research (3, 21). In the study of Celik et al., mothers exhibited high levels of anxiety when their children had frequent seizures was significantly higher compared to mothers whose children have infrequent seizures, during the pandemic. However, no statistical difference was observed between mothers of CAWE who attended their appointment during the pandemic and those of the mothers who did not attend their follow-up appointment (27). On the contrary, a study in Sri Lanka showed low levels of stress among caregivers of CAWE during COVID-19 (11). However, parents in our study were more worried that they could not provide for their children and they expressed their worry that their children were missing experiences due to the lockdown. This is an aspect that has not yet been addressed in current literature besides the survey of the University of Pittsburg, according to which, family caregivers reported an increased responsibility during the COVID-19, related to interrupted access to care, resulting in lifestyle adjustments, increased worry, fear, depression and anxiety (11, 14). It appears that the COVID-19 restrictions challenged the parental role as providers and caregivers further burdening their parental self-perception (11, 22).

Parents have also acknowledged the “silver linings” during the lockdown: they found themselves less worried as they could monitor their child constantly and prevent an eventual seizure. Although current research agrees on the positive outcomes that parents make of their experience of having a CAWE (11, 23), this findings reveal a tendency of overprotection which is common as parental practice among parents with children with chronic diseases (25). Thus, the restrictive measures might have exacerbated this attitude.

Despite the interest of this study, some limitations may be highlighted. Firstly, fathers are under-represented in this study. Similar to other studies, fathers' experiences are rare to obtain. Furthermore, the varying seizure characteristics of children of parents interviewed and the recruitment paths are chosen, the present sample cannot be considered representative of all parents of children living with epilepsy (28).

### 4.1. Implications for practice

In previous research, it has been largely established that parents of CAWE experience increased levels of stress, concerns, and depressive symptoms but also reported their experienced post-traumatic growth on a personal, parental and family level (16, 25, 29, 30). Our research adds to current literature, as it explores the experiences of parents of CAWE during a severe lockdown where their parental role was challenged. Thus, the need for more evidence-based research and interventions is pointed out in order for parents to work on their parental role and enhance their understanding of their child's epilepsy condition so they could gain greater confidence in their role. Finally, parent groups with CAWE where families have the opportunity to share the challenges they encountered could also enhance their confidence and add to self-management strategies on a psycho-emotional level.

## 5. Conclusion

In general, our findings emphasize the vulnerability of the parental / caregiver role of CAWE in the context of the COVID-19 pandemic. Moreover, specific practices could be proposed, e.g., supporting these families that are often overwhelmed by their role. Additionally, these results could be adopted as indicators to improve the quality of care for both young patients with epilepsy and their caregivers.

## Data availability statement

The raw data supporting the conclusions of this article will be made available by the authors upon reasonable request.

## Ethics statement

The studies involving human participants were reviewed and approved by Ethics Committee of the National and Kapodistrian University of Athens. The patients/participants provided their written informed consent to participate in this study.

## Author contributions

FK and MA: conceptualized the research design, developed the data collection tools, collected data, analyzed data, drafted the manuscript, and undertook critical review of the manuscript and final approval.

## Footnotes

^1^COVID-19 stands for coronavirus disease.

^2^This participant used the word “house” in their narratives as opposed to an apartment. A house is usually larger and more spacious.
