# Corrigendum: COVID-19 and parents of children with epilepsy: experiences and positive changes

**DOI:** 10.3389/fpubh.2023.1209062

**Published:** 2023-05-10

**Authors:** Flora Koliouli, Marianna Andrianakou, Philia Issari

**Affiliations:** ^1^Psychology Department, National and Kapodistrian University of Athens, Athens, Greece; ^2^School of Early Childhood Education, Aristotle University of Thessaloniki, Thessaloniki, Greece

**Keywords:** parents, children with epilepsy, COVID-19, lived experiences, qualitative research

In the published article, there was an error in the author list, and author “Philia Issari” was erroneously excluded. The corrected author list appears below.

“Flora Koliouli^1,2^, Marianna Andrianakou^1^ and Philia Issari^1^

The corrected citation appears below.

“Koliouli F, Andrianakou M and Issari P (2023) COVID-19 and parents of children with epilepsy: experiences and positive changes. *Front. Public Health* 11:1079518. doi: 10.3389/fpubh.2023.1079518”

The corrected **Author contributions** Section appears below.

“FK, MA, and PI: conceptualized the research design, developed the data collection tools, collected data, analyzed data, drafted the manuscript, and undertook critical review of the manuscript and final approval.”

The authors apologize for this error and state that this does not change the scientific conclusions of the article in any way. The original article has been updated.

